# Focused Ultrasound Simultaneous Irradiation/MRI Imaging, and Two-Stage General Kinetic Model

**DOI:** 10.1371/journal.pone.0100280

**Published:** 2014-06-20

**Authors:** Sheng-Yao Huang, Chia-En Ko, Gin-Shin Chen, I-Fang Chung, Feng-Yi Yang

**Affiliations:** 1 Institute of Biomedical Informatics, National Yang-Ming University, Taipei, Taiwan; 2 Center for Systems and Synthetic Biology, National Yang-Ming University, Taipei, Taiwan; 3 Department of Biomedical Imaging and Radiological Sciences, National Yang-Ming University, Taipei, Taiwan; 4 Biophotonics and Molecular Imaging Research Center, National Yang-Ming University, Taipei, Taiwan; 5 Division of Medical Engineering Research, National Health Research Institutes, Miaoli County, Taiwan; 6 Department of Diagnostic Radiology, Mackay Memorial Hospital of Tan-Shui, Taipei, Taiwan; Italian Institute of Technology, Italy

## Abstract

Many studies have investigated how to use focused ultrasound (FUS) to temporarily disrupt the blood-brain barrier (BBB) in order to facilitate the delivery of medication into lesion sites in the brain. In this study, through the setup of a real-time system, FUS irradiation and injections of ultrasound contrast agent (UCA) and Gadodiamide (Gd, an MRI contrast agent) can be conducted simultaneously during MRI scanning. By using this real-time system, we were able to investigate in detail how the general kinetic model (GKM) is used to estimate Gd penetration in the FUS irradiated area in a rat's brain resulting from UCA concentration changes after single FUS irradiation. Two-stage GKM was proposed to estimate the Gd penetration in the FUS irradiated area in a rat's brain under experimental conditions with repeated FUS irradiation combined with different UCA concentrations. The results showed that the focal increase in the transfer rate constant of *K*_trans_ caused by BBB disruption was dependent on the doses of UCA. Moreover, the amount of *in vivo* penetration of Evans blue in the FUS irradiated area in a rat's brain under various FUS irradiation experimental conditions was assessed to show the positive correlation with the transfer rate constants. Compared to the GKM method, the Two-stage GKM is more suitable for estimating the transfer rate constants of the brain treated with repeated FUS irradiations. This study demonstrated that the entire process of BBB disrupted by FUS could be quantitatively monitored by real-time dynamic contrast-enhanced magnetic resonance imaging (DCE-MRI).

## Introduction

This paper offers improvements in the use of focused ultrasound (FUS) for non-invasive medical treatment [1], in particular the diagnosis and treatment of brain tumors without associated tissue damage. FUS has been accepted for the past decade or so as a potential form of effective non-invasive medical treatment [2] for conditions including brain tumors. The brain is protected by a blood-brain barrier (BBB), which is a tight layer of endothelial cells surrounding the brain which is not permeable, and hence requires incisions to introduce drugs or diagnostic material into the brain tissue. When used with an ultrasound contrast agent (UCA), pressure produced by FUS acts upon the microbubbles in the UCA causing them to implode and produce cavitation which results in a precisely controlled rupture of the endothelial tissue, disrupting the brain's blood-brain barrier (BBB) [3]–[6], and allowing macromolecular drugs to infiltrate into the lesion area through an opening produced from rupture of the tight junction, thereby facilitating treatment [7]–[9].

However, different FUS acoustic pressures and UCA concentrations can lead to different degrees of the BBB disruption and thus have different effects on the BBB [4], [10]–[12], including the possibility of tissue damage. Therefore, there have been many studies in recent years focusing on how to select the appropriate acoustic parameters, UCA size, or UCA concentration in order to most effectively disrupt the BBB [13]. Many studies have sought to estimate the degree of the BBB disruption by using the kinetic model for analysis of dynamic contrast-enhanced magnetic resonance imaging (DCE-MRI) [13], [14]. This method first requires using DCE-MRI with an MRI contrast agent (Gadodiamide (Gd)) to observe changes in brain images before and after FUS irradiation. Subsequently, analysis of the brain tissue is conducted to measure Gd penetration into the brain tissue surrounding the blood vessels of the FUS irradiation region as a result of the BBB disruption. This is done through the use of image analysis and the kinetic model. Finally, the degree of the BBB disruption is described in a quantitative manner by assessing the change in parameters of the kinetic model. Vlachos *et al*. have pointed out that the degree of the BBB disruption increases with a larger microbubble or a higher level of FUS power (i.e., resulting in an increase in the amount of Gd penetration) [13].

The most commonly used kinetic model is the general kinetic model (GKM), proposed by Tofts *et al*. in 1999 [15], which mainly adopts two transfer rate constants, *K*_trans_ and *K*_ep_, to describe the changes in Gd concentration in blood vessels and the FUS irradiated region of the brain. *K*_trans_ and *K*_ep_ represent the transfer rate constants from the blood vessels to the extravascular extracellular space (EES) and from the EES to the blood vessels, respectively. The *K*_trans_ value for the normal BBB is approximately zero. However, once the BBB is disrupted, the *K*_trans_ values will increase within a very short period of time due to the increased permeability between the cerebral blood vessels and cerebral tissue [13], [14]. Furthermore, studies of cancer treatment with FUS irradiation have reported that brain tumors have the relatively weak BBB [16]–[18], resulting in a higher *K*_trans_ value. After treatment of the tumors in these studies, the relative value of *K*_trans_ was reduced. Therefore, the *K*_trans_ value can be considered a good indicator of the degree of the FUS-induced BBB disruption.

Although the objective of this study was also to assess disrupting the BBB by using the GKM on analysis of DCE-MRI as done by previous studies [13], [14], [19], here our approaches have two novel characteristics. First, we established a real-time system for the FUS-induced BBB disruption experiments, whereby the MRI- compatible ultrasound transducer and the animals both entered the MRI machine. This system provided for both UCA and Gd injections and for FUS irradiations and image acquisitions. This system allows for a better analysis without interference from the time delay caused by having to remove the rat to perform injections and FUS irradiation, because the time delay can result in changes to the BBB, affecting analysis, as in past studies. Moreover, the number of FUS rounds and the intensity of the FUS beams, as well as the concentration of UCA injections, can also be adjusted according to the changes in permeability in the BBB. In addition, in the repeated FUS irradiation experiments in this real-time environment, the assessment of the BBB disruption and the estimation of transfer rate constants in different stages of the experiments could not be accurately evaluated by using the GKM (in previous studies, the GKM was only used to assess the single FUS-induced BBB disruption). Therefore, we updated the equation for GKM and proposed Two-stage GKM for the estimation of transfer rate constants in the experiment with two rounds of FUS irradiation. This method can be further applied in the estimation of transfer rate constants in an experiment with multiple rounds of FUS irradiation, while also improving FUS treatment and avoiding risks associated with it.

## Materials and Methods

### Animal Preparation

A total of twenty-one 7-week-old male Sprague-Dawley (SD) rats (12 rats for FUS irradiation and DCE-MRI process, and 9 rats for FUS irradiation and Evans blue extraction), ranging from 280 g to 300 g in weight, were used in strict accordance with the recommendations in the Guide for the Care and Use of Laboratory Animals of the National Yang-Ming University. The protocol was approved by the Committee on Institutional Animal Care and Use Committee (IACUC) of the National Yang-Ming University (Permit Number: 1001228). The rats were anesthetized intraperitoneally with urethane (1.2 g/kg) prior to performing experiments. For FUS irradiation and DCE-MRI process, the scalps of the rats were shaved to facilitate the use of bregma of each rat's skull as a reference point.

### Ultrasound Experimental Setup

**Figure 1:**
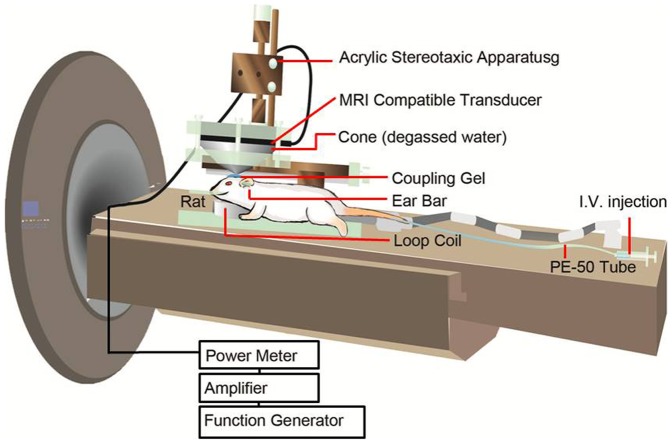
Schematic diagram of the real-time MRI for monitoring the focused ultrasound-induced BBB disruption.

Our real-time system for the FUS-induced BBB disruption experiments is shown in Fig. 1. The MRI-compatible ultrasound probe used was a single-element transducer for focused ultrasound (H-101, SONIC CONCEPTS, INC) with a diameter of 64 mm, a curvature radius of 62.64 mm, and a center frequency of 1.04 MHz. The transducer was mounted with a removable cone filled with degassed water and a tip capped with a polyurethane membrane. The FUS focal point was positioned approximately 5 mm below the central point of the polyurethane membrane. The FUS probe was moved to anchor point, the position of 3.5 mm posterior and 3.5 mm right lateral to the bregma via the stereotaxic apparatus. The coupling gel was placed between the rat's head and the polyurethane membrane located at the circular hole of the probe. The intravenous catheter was inserted into the lateral tail vein of the rat and then attached to a PE-50 extension tubing prefilled with heparinized saline. Next, the acrylic stereotaxic apparatus with the fixated rat and the probe were placed on the scanner table of a 3T MRI machine (TRIO, 3T MRI, Siemens Magneton, Germany) with a loop coil of approximately 4 cm in diameter used for radio frequency reception. The receive-only coil was placed under the rat's head and then positioned via the laser positioning system for 3T MRI. The details for the experimental ultrasound setup were the same as those used in our previous study [20].

Note that, the waveform generator (33220A, Agilent Inc., Palo Alto, USA) connected to the power amplifier (500–009, Advanced Surgical Systems, Tucson, AZ) drove the transducer to generate the pulse wave signal. A power sensor/meter (4421, Bird, Cleveland, OH, USA) was used to measure the actual input of electric power. The focal spot (−6 dB) of the ultrasound transducer was 1.5 mm in width and 8 mm in depth. The parameters of the ultrasound were burst length 50 ms, duty cycle 5%, pulse repetition frequency 1 Hz, ultrasonic irradiation time 60 s, and acoustic power 4.1 W. The administration of UCA was SonoVue (Bracco International, Amsterdam, Netherlands), which contains microbubbles with a mean diameter of 2.5 µm at a concentration of 1−5×10^8^ bubbles/mL. The UCA was injected into the tail veins of the rats in one or both of two doses, 150 µl/kg and 450 µl/kg, depending on the experiment.

### Three Sets of Experiments

Three different sets of experiments were performed based on the UCA concentration and the rounds of FUS treatment: Single 150, Single 450, and Repeated 150/450. The 150 µl/kg doses of UCA along with a single treatment of FUS were applied in the Single 150 experiment, whereas the 450 µl/kg UCA dose and a single treatment of FUS were used in the Single 450 experiment. In the Repeated 150/450 experiment, a first treatment of FUS occurred after the 150 µl/kg UCA dose and a second treatment of FUS occurred following the 450 µl/kg dose.

### DCE-MRI Protocol

**Figure 2:**
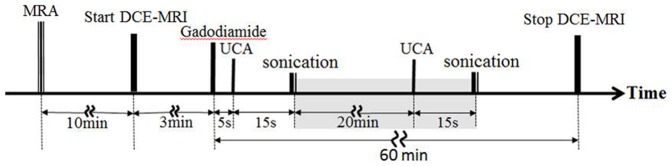
Time line for the BBB disruption experiments. Only for the Repeated 150/450 experiment, the shaded part was included in the experimental process. All experiments were performed on the MRI scanner.

As shown in Fig. 2, a set of 3D TOF-MRA (Time-of-Flight magnetic resonance angiography) images were first obtained by DCE-MRI in order to monitor the position of the middle cerebral artery (MCA) in the rat's brain [21]. The parameters of the TOF-MRA were the number of slices  = 160, TR/TE  = 14/5.42 ms, flip angle  = 20°, slice thickness  = 0.3 mm, FOV  = 85×85 mm^2^, matrix  = 256×256, slab = 5, and scanning time  = 10 min 27 s.

Subsequently, 40 sets of T_1_-weighted DCE-MRI images were made in order to assess the changes in the BBB disruption in the rat's brain prior to and after ultrasound irradiation (i.e., through observation of the permeability of MRI contrast agent (Gd) surrounding the irradiated brain region). These 40 sets of DCE-MRI images consisted of (a) the two sets of MRI images prior to Gd injection (scanning about 3 minutes) referred to as the initial condition of the brain image and (b) a total of 38 sets of DCE-MRI images acquired continuously for 1 hour after the injections of Gd and UCA and following FUS irradiation. The parameters for each set of T_1_-weighted DCE-MRI images were the number of slices  = 22 (covering the entire brain in order to detect the location of the BBB disruption), TR/TE  = 500/13 ms, FOV  = 47×8 mm^2^, matrix  = 152×256 pixels, slice thickness  = 1.5 mm, and scanning time  = 97 s.

Note that, for the Single 150 and Single 450 experiments, the rats were first injected Gd (1 mmol/kg) from the tail vein with a PE-50 tubing prior to the third sets of MRI and then were additionally injected with UCA 5 seconds later. After a 15 second pause for the diffusion of UCA into the blood vessels of the rat's brain, the right cerebral hemisphere of the rat's brain was irradiated by FUS. For the Repeated 150/450 experiment, the second injection of UCA was conducted 20 minutes after the start of the first FUS. Fifteen seconds after UCA injection, the right cerebral hemisphere of the rat's brain received the second round of FUS (as shown in the shaded area of Fig. 2).

### MRI Data Acquisition and Signal Analysis

Similar to previous studies, the research here determined the transfer rate constants *K*_trans_ and *K*_ep_ by analyzing the T_1_-weighted DCE-MRI images and adopting GKM calculation, thereby evaluating the differences in the BBB disruption according to different UCA injection concentrations and the number of FUS irradiations. Predicting the transfer rate constants *K*_trans_ and *K*_ep_ of GKM requires knowledge of the changes in Gd concentration in the brain tissue and cerebral artery receiving FUS irradiation.

The location of the cerebral artery was determined from one of the 3D TOF-MRA images [22], [23]. The corresponding slice in each set of DCE-MRI images was then used to observe the Gd concentration of the cerebral artery. Due to the Gd injection and FUS irradiation, there was a rapid increase in the Gd concentration of the cerebral artery, after which the concentration was gradually reduced until it reached a fixed value [24]. Therefore, the corresponding DCE-MRI image signals of the 40 points in time at the cerebral artery position should reflect changing trends in Gd concentration. The identified DCE-MRI image signals shall be further transformed (further elaborated at the GKM portion later on) to represent the changes in Gd concentration in the cerebral artery.

Estimation of the Gd concentration in the brain tissue region receiving FUS irradiation required the following steps. First, 3 consecutive slices from a set of DCE-MRI images were selected to monitor the FUS irradiation area of the rat's right brain. Subsequently, each of the 3 consecutive DCE-MRI images was set to the region of interest (ROI) of 25 pixels at brighter portions of the FUS irradiated region of the right brain. After determining the average strength of the image signals at each of the 3 ROI, the mean strength of the three averages taken together was calculated to represent the status of the FUS irradiated region in the right brain tissue at a point in time at which Gd penetrated into the tissues as a result of the BBB disruption. Finally, the respective mean values from the corresponding DCE-MRI image signals of the 40 points in time were obtained through the above two steps and then a transformation (elaborated at the GKM portion later on) was used to represent the changes in Gd concentration in the right brain tissue with FUS irradiation. Similarly, 3 ROI with the same size (3 consecutive slices corresponding to the same slices for the right brain) were selected at each point in time in regions of the left brain which were mirror images of the ultrasound focused areas in the right brain. The mean value of the signal strengths of the three ROI was also calculated, as mentioned above for the right brain, to represent the status of the left brain tissue at a point in time at which Gd could not penetrate into the tissues due to the BBB.

### General Kinetic Model

The blood flow through the blood vessels, the permeability of the brain capillaries, and other related physiological constants (e.g., *K*_trans_ and *K*_ep_) can be properly estimated by using the kinetic model on the analysis of the DCE-MRI images [10], [25]. The general kinetic model (GKM) [13], [14] was widely used as a reference for the changes seen in the spread of the Gd concentration in the brain tissue and cerebral artery upon BBB disruption. The brief description of the GKM is given as follows.

In the GKM, *C_p_* (t) and *C_t_* (t) represent the Gd concentration at time *t* for the cerebral artery and the FUS irradiated brain tissue region, respectively. They are expressed as follows [26], [27]: 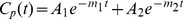
(1)

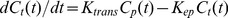
(2)


Here *A*_1_, *A*_2_, *m*_1_, *m*_2_, *K*_trans_, and *K*_ep_ are constants. Substitute Eq. (1) into Eq. (2) and the following formula is obtained: 
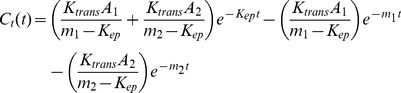
(3)


The values of the four constants in Eq. (1) can be properly estimated by using the data regarding the Gd concentration of the cerebral artery over 40 points in time. *K*_trans_ and *K*_ep_ values can then be assessed from the data regarding the Gd concentration for the brain tissue region with FUS irradiation over the same 40points in time. Note that the Levenberg-Marquardt fitting algorithm in Matlab is used for estimation of those variable values. The Gd concentration in Eqs. (1) and (2) can be obtained from the transformation of DCE-MRI image signals using the formula below [28]: 
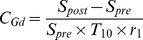
(4)


Here, 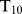 is the 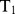 relaxation time of the arterial blood or the brain tissue before the Gd administration. T_10_ = 0.9 s is used for the brain tissue area, whereas T_10_ = 1.5 s is used for the cerebral artery [28]. r_1_ is the DCE-MRI relaxivity of the contrast, using the reference value of the 3T MRI system r_1_ = 4.62 mM^−1^s^−1^ [26]. *S*_pre_ and *S*_post_ represent the image signal values before and after Gd injection, respectively [27]. In addition, as most of the Gd would accumulate in the plasma (plasma constitutes 55% of the blood volume), the value obatined from Eq. (4) is multiplied by 0.55 in the calculation of the Gd concentration for the cerebral artery [13], [29].

### Two-stage GKM

In this study, we proposed a Two-stage GKM approach to monitor the changes in permeability resulting from FUS radiation (with different UCA concentration) for the Repeated 150/450 experiment, as follows.

**Figure 3.The:**
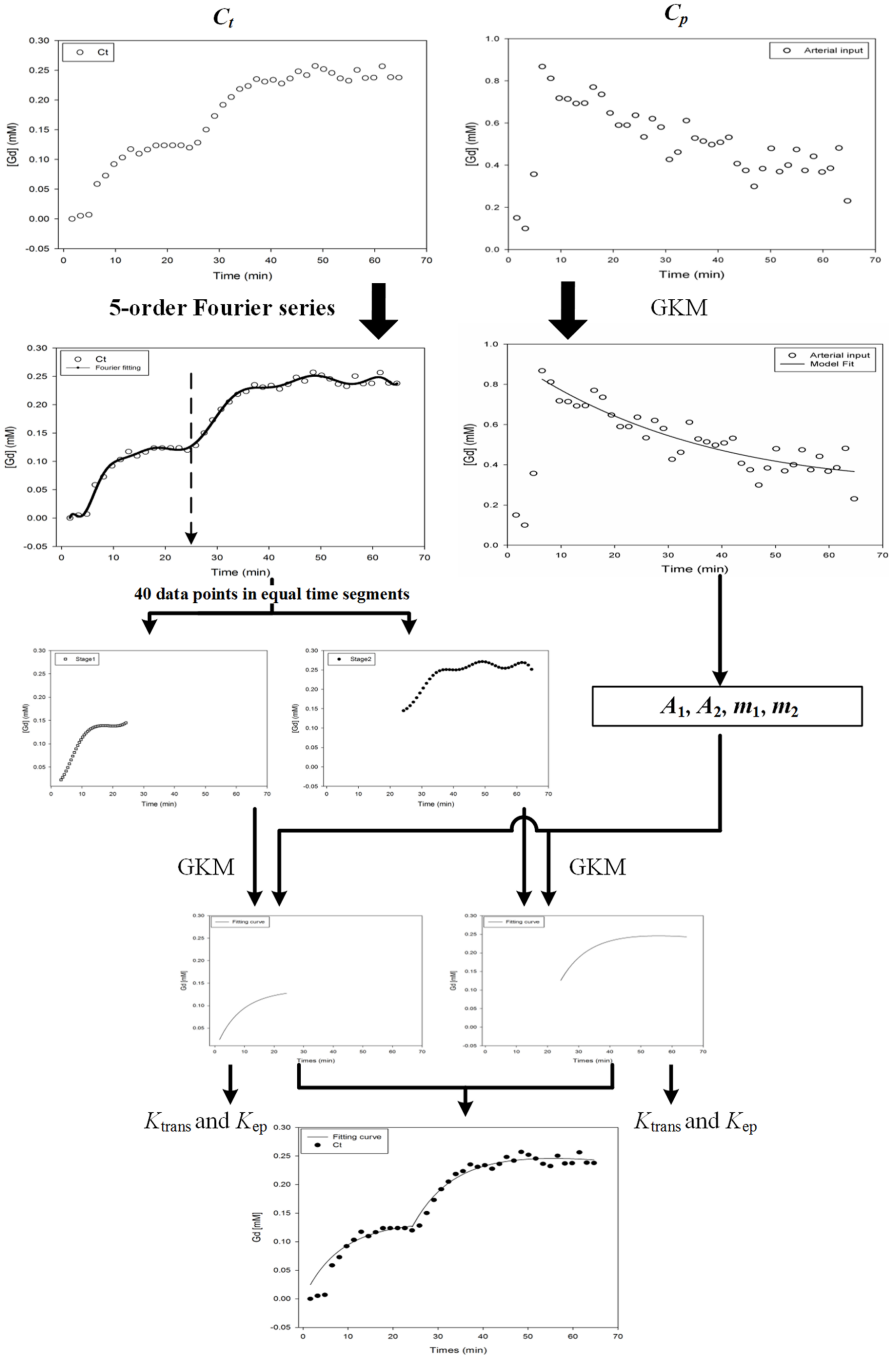
flow diagram of the Two-stage GKM process.

As shown in Fig. 3, first we still used all of the yielded data points for the Gd concentration in the cerebral artery (*C_p_*) to estimate the values of four parameters used in representation of the *C_p_*(t) function (Eq. (1) in GKM). Next, we adopted a strategy to correctly estimate the trend of the data points for Gd concentration in the FUS irradiated brain tissue region (*C_t_*) using GKM as follows: (1) the curve to estimate the trend of the data points for original Gd concentration in the FUS irradiated brain tissue region (*C_t_*) was first produced using the Fourier function in MATLAB (here a 5-order Fourier series was adopted); (2) we then used the second FUS irradiation as the point in time at which to separately segment the curve for *C_t_* into two parts; (3) based on the parameters of the curve function obtained from this process, data points in each of the two parts were selected by dividing the part into 40 equal time segments. (4) the corresponding *K_trans_* and *K_ep_* in each part were separately estimated using Eq. (3) in GKM, the two sets of synthetic data points for C*_t_*, and the four parameters for C*_p_*. Note that, the curve to estimate the C*_t_* trend can also be produced using the linear approach or the nearest neighbor approach.

### Evans Blue Extraction

The experimental portion for the Evans Blue (EB) extraction adopted the Single 150, Single 450 and Repeated 150/450 experimental conditions and used 3 rats for each experiment. Each rat was injected with EB in its tail, followed by UCA after 5 s and FUS irradiation on the brain tissue after 15 s. Under the Repeated 150/450 experimental condition, the second UCA injection and second FUS irradiation were conducted 20 min after the first FUS irradiation. The procedures for EB extraction were detailed in our previous report. The quantity of EB was expressed as Mean ± SEM and then analyzed using the t-test to check for statistical significance.

## Results

**Figure 4:**
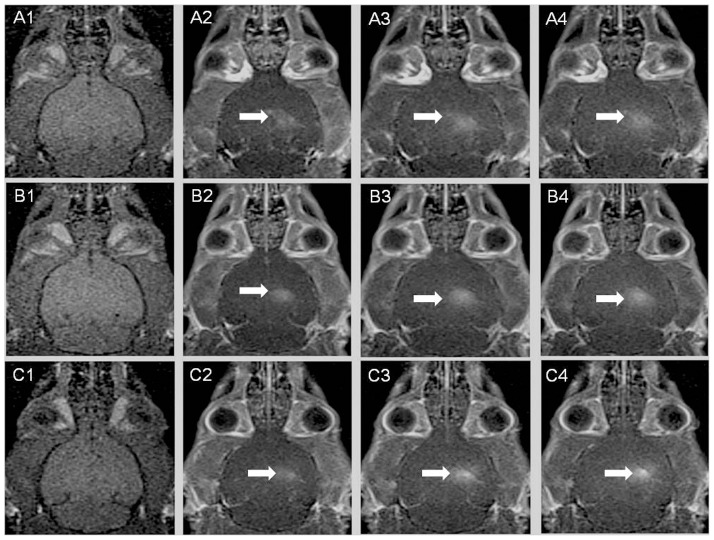
Illustration of the T1 DCE-MRI images of one rat each for three sets of experiments: (A) Single 150; (B) Single 450; and (C) Repeated 150/450. The first column of panels for (A), (B) and (C) shows the DCE-MRI images before the Gd injection; the second to fourth columns of panels for (A) and (B) show the images at 20, 40, and 60 min after FUS irradiation; the second panel for (C) shows the image at 20 min after the first FUS irradiation; The third and fourth panels for (C) show the images at 20 and 40 min after the second FUS irradiation.

For the three sets of experimental conditions (Single 150, Single 450, and Repeated 150/450), Fig. 4 shows the DCE-MRI images of the brain of a single rat. The extraction of the DCE-MRI images here is based on the same cross sectional structure of the brain. The white region in the figure represents the penetration after Gd diffuses into the brain tissue following the BBB disruption as a result of FUS irradiation. As there was no FUS irradiation of the left hemisphere of the brain, the DCE-MRI images of this portion display no white areas, whereas in contrast the images from the right hemisphere of the brain display obvious white areas due to FUS irradiation.

In addition, Fig. 4 displays the DCE-MRI images of one rat each for three sets of experiments at different points in time. (See the caption for Fig. 4 for more detail). We further observed that the hyperintense regions become more pronounced (from left to right), meaning that the amount of Gd penetration increased with longer periods of FUS irradiation. Besides, it was also observed that the hyperintense portion (from top to bottom) became more pronounced due to increases in the concentration of the UCA injection.

**Figure 5:**
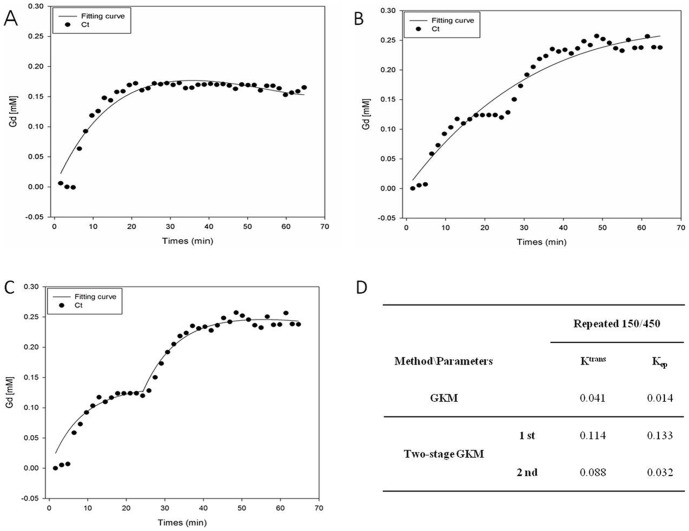
Estimation of the changes in Gd concentration using GKM or Two-stage GKM. (A) estimation of the changes in Gd concentration at the right hemisphere of the rat's brain tissue for the single FUS irradiation experiment using GKM; (B) estimation of the changes in Gd concentration in the right hemisphere of the rat's brain tissue for the repeated FUS irradiation experiment using GKM; (C) based on the condition in (B), estimation of the changes in Gd concentration in the right hemisphere of the rat's brain tissue for the repeated FUS irradiation experiment using Two-stage GKM; (D) list of the permeability values K_trans_ and K_ep_ estimated from (B) and (C). The data points in the figure show the changes in Gd concentration with time in the right hemisphere of the rat's brain tissue due to FUS irradiation. The curve was estimated using GKM or Two-stage GKM.

Figs. 5A–5C show the trend for the Gd concentration signals on the right side of the brain tissue of rats under different experimental conditions. The vertical axis represents the Gd concentration, the horizontal axis represents the time (40 points in time), the data points represent the measured Gd concentration, and the curve is estimated through GKM or Two-stage GKM. Figs. 5A and 5B show the Gd concentration signals on the right side of a rat's brain tissue estimated using GKM under the Single 150 and Repeated 150/450 experimental conditions, respectively. From these two figures, it can be seen that GKM was able to appropriately estimate the changes in Gd concentration in the brain tissue for the single FUS irradiation experiment. However, GKM was unable to accurately estimate the changes in Gd concentration in the brain tissue for the repeated FUS irradiation experiment (in particular, it cannot reflect the changes in Gd concentration in the brain tissue after the second FUS irradiation). Fig. 5C used the Gd concentration signals of the same rat as Fig. 5B but conducted estimation in stages using Two-stage GKM. Fig. 5C clearly shows that Two-stage GKM in stages could appropriately estimate the changes in Gd concentration in the brain tissue for the repeated FUS irradiation experiment. Fig. 5D shows the transfer rate constants *K*_trans_ and *K*_ep_ obtained via GKM in Fig. 5B or Two-stage GKM in Fig. 5C, respectively. When the value of *K*_trans_ is used as an indicator to describe the BBB disruption under FUS irradiation, the results in Fig. 5D show that GKM underestimated the *K*_trans_ value for the Repeated 150/450 experiment and was unable to show the impact brought about by repeated FUS irradiation. Two-stage GKM, however, showed the impact of the first and second FUS irradiation respectively using two *K*_trans_ values.

**Figure 6:**
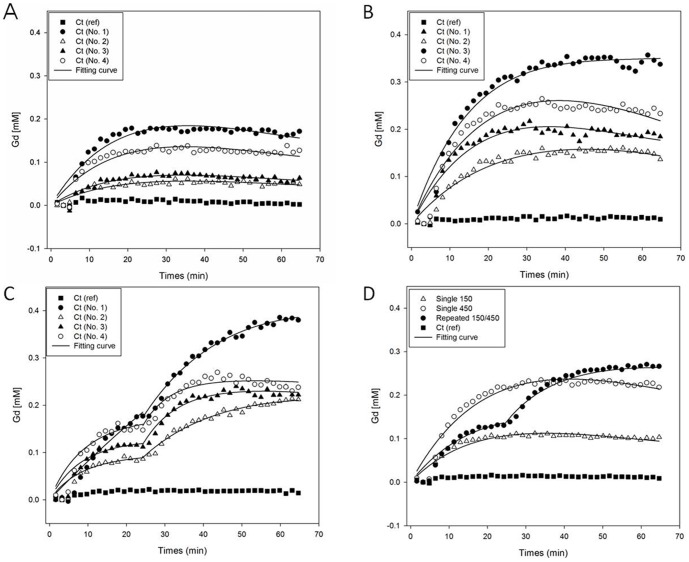
Estimation of the changes in Gd concentration in the right hemisphere of the brain tissue of four rats for both Single 150 and Single 450 experiments using GKM, as well as estimation of the changes in Gd concentration in the right hemisphere of the brain tissue of four rats for the Repeated 150/450 experiment using Two-stage GKM. (A) Single 150; (B) Single 450; (C) Repeated 150/450; (D) estimation of the mean changes in Gd concentration in the brain tissue for the various groups under the 3 experimental conditions.

Figs. 6A–6C further depict that GKM was able to appropriately estimate the changes in Gd concentration on the right side of the brain tissue of 4 rats for both Single 150 and Single 450 experiments, and Two-stage GKM was also able to appropriately estimate the changes in Gd concentration on the right side of the brain tissue of four rats for the Repeated 150/450 experiment. Each sub figure in Figs. 6A–6C has 5 estimated curves corresponding to the 5 sets of data points. Amongst them, the set of data points with the lowest Gd concentration is the mean changes in Gd concentration on the left side of the brain tissue of 4 rats, which is used as the reference point to modulate Gd concentration at the FUS irradiation region of each rat. The other 4 sets of data points represent the different changes in Gd concentration on the right side of the brain tissue of 4 rats due to FUS irradiation under a specific experimental condition. In addition, Fig. 6D shows the estimation of the mean changes in Gd concentration in the brain tissue for the various groups under 3 sets of experimental conditions. The set of data points in Fig. 6D with the lowest Gd concentration is the mean changes in Gd concentration on the left side of the brain tissue of 12 rats. It is used as the reference point to modulate the mean changes in Gd concentration in the right hemisphere of the brain tissue of 4 rats for each experiment. Each set of the other 3 sets of data points in Fig. 6D represents the mean changes in Gd concentration in the right hemisphere of the brain tissue of 4 rats in a specific experiment due to FUS irradiation. It can be seen from Fig. 6D, that the estimated curve for the changes in Gd concentration under the Repeated 150/450 experimental condition were rising slightly faster than that under the Single 150 experimental condition (during the first twenty minutes this non-significant difference is likely due to the use of four different rats for each experiment). The rising trend of Gd concentration for the Repeated 150/450 experiment changed again after the second FUS injection. Eventually, the Gd concentration for the Repeated 150/450 experimental condition would be higher than that of the Single 450 experimental condition. Table 1 shows the mean permeability coefficients *K*_trans_ and *K*_ep_ using GKM or Two-stage GKM, as well as *v*_e_ = *K*_trans_/*K*_ep_ under the Single 150, Single 450, and Repeated 150/450 experimental conditions. When the value of *K*_trans_ is again used as an indicator to determine the BBB disruption due to FUS irradiation, the results in Table 1 show that FUS irradiation under the Single 450 experimental condition could lead to a more significant BBB disruption compared to the Single 150 experiment. It could also be seen that using GKM to conduct analysis under the Repeated 150/450 experimental condition would lead to underestimation of the *K*_trans_ value (even smaller than that of the Single 450 experimental condition). On the other hand, Two-stage GKM used the two *K*_trans_ values to show the impact of the first and second FUS irradiation under the Repeated 150/450 experimental condition.

**Table 1:** The mean transfer rate constant computed using GKM or Two-stage GKM under Single 150, Single 450, and Repeated 150/450 experimental conditions.

	GKM			Two-stage GKM	
	Single 150 (n = 4)	Single 450 (n = 4)	Repeated150/450 (n = 4)	Repeated 150/450 (n = 4)	
Right hemisphere				1st	2nd
*K_trans_* (min^−1^)	0.059±0.009	0.126±0.028	0.067±0.009	0.093±0.011	0.068±0.005
*K_ep_* (min^−1^)	0.049±0.012	0.044±0.009	0.012±0.001	0.053±0.001	0.015±0.005
*v_e_*				1.75	4.53
	1.2	2.86	5.58	3.6*	
Left hemisphere					
*K^mean^_trans_* (min^−1^)			0.012±0.002		
*K^mean^_ep_* (min^−1^)			0.093±0.031		

The ratio of time in the two stages of the Two-stage GKM is 1st (20 min): 2nd (40 min). *Using a weighted mean calculation based on time period for each segment would yield *v_e_* = (1.75*1+4.53*2)/3 = 3.6.

**Figure 7:**
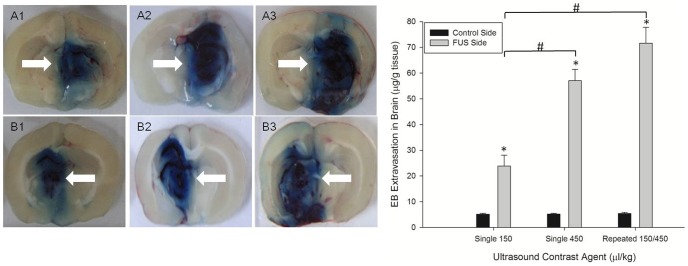
The brain slices of Evans blue extravasation. The upper and lower half sub figures in the left figure show the front and back of the sonicated brain divided into slices respectively. It is arranged in order from left to right according to the Single 150, Single 450, and Repeated 150/450 experimental conditions respectively. The figure on the right shows the quantitative data for the Evans blue penetration (*and# implies that p<0.05; n = 3).

Fig. 7 shows the results of the EB experiment. EB clearly penetrated into the area in the right hemisphere of the rat's brain subjected to FUS irradiation. In addition, more drastic permeability was also seen (larger BBB disruption) for the Single 450 and Repeated 150/450 experimental conditions. The right subfigure of Fig. 7 shows the measured amount of EB extravasation. The vertical axis is the extracted amount of EB, and horizontal axis represents the three experimental conditions of Single 150, Single 450, or Repeated 150/450 (3 rats were used in each experiment). It is clearly observed that for the 3 different experiments there is a very small amount of EB and not much of a difference in the amount of EB in the left hemisphere of the rat's brain due to the intact BBB. However, the amount of EB penetrating the right hemisphere of the rat's brain under the Single 150 experimental condition is less than that of the Single 450 experimental condition, which is in turn less than that of the Repeated 150/450 experimental condition. The mean amount of EB penetrating the tissue in the right hemisphere of the rat's brain was 23.85* µ*g/g tissue (Single 150), 57.08* µ*g/g tissue (Single 450), and 71.56* µ*g/g tissue (Repeated 150/450).

## Discussion

Previous studies compared single FUS irradiation with repeated FUS irradiation of the rat brain, finding that repeated FUS irradiation caused increased EB penetration of the brain tissue due to further BBB disruption [19], [29]. In addition, previous research [30] had also *in vivo* illustrated that multiple FUS irradiations shall result in a larger degree of BBB disruption. Furthermore, Park *et al*. [29] adopted the DCE-MRI technique to observe the change of Gd penetration and then only showed the estimated *K_trans_* values by GKM after performing the FUS irradiations.

In this study, we further used GKM in conjunction with single FUS irradiation and proposed Two-stage GKM in conjunction with repeated FUS irradiation for analysis of changes in DCE-MRI brain images of the rat before and after FUS irradiation, to explore the relationship between Gd penetration of the brain tissue and irradiation with single and repeated FUS. We also verified the correlation between the experimental results through analysis of DCE-MRI images and the amount of *in vivo* penetration of EB in the FUS irradiated area in a rat's brain under single and repeated FUS irradiation.

To verify that the proposed Two-stage GKM method is more suitable for repeated FUS irradiation experiments than the GKM method [31], this study used the following three methods. Because the trend in Gd concentration changed significantly between the first FUS irradiation and the second FUS irradiation, we first used Two-stage GKM to estimate the changes in Gd concentration in the brain tissue in stages for the repeated FUS irradiation. The results presented in Figs. 5 and 6 show the proper data estimation in stages for the repeated FUS irradiation experiment, show that GKM is only suitable for use in the single FUS irradiation experiment, and finally that with higher concentrations of UCA for the single FUS irradiation experiment more Gd penetration to the region of the brain tissue where FUS irradiation occurs. Notably, in the repeated FUS irradiation experiment, Two-stage GKM was used to estimate the changes in the Gd concentration in the brain tissue, but GKM was used to estimate the change in the Gd concentration in the blood vessels of the brain. The primary reason for this is that the decrease in Gd concentration for the blood vessels in the brain did not produce a clear enough change in the trend due to the second FUS irradiation.

Furthermore, it has been demonstrated that the permeability coefficient *K*_trans_ in GKM can be a good indicator for the BBB disruption under FUS irradiation. Therefore, Table 1 shows that the *K*_trans_ value obtained under different experimental conditions not only can be used to show that higher UCA concentration produced more disruption of the BBB in the single FUS irradiation experiments, but it can also indicate the difference in BBB disruption between the first and second FUS irradiation by using Two-stage GKM for the repeated FUS irradiation experiment.

Previous studies have proposed that there is a direct relationship between the *v*_e_ = *K*_trans_/*K*_ep_ value [15] and the quantity of materials in the blood vessel that penetrate into the brain tissue due to BBB disruption. As shown in Results, the proportional relationship in the amount of EB penetrating the right hemisphere of the rat's brain among Single 150, Single 450, and Repeated 150/450 is 0.33∶0.8∶1, which is almost an exact match with the ratio of the *v*_e_ values in Table 1 (1.2: 2.86: 3.6≅0.33: 0.79: 1) in the rat's right brain under these three experimental conditions (the *v*_e_ value for the Repeated 150/450 experiment was obtained by using Two-stage GKM). However, when GKM was used to estimate the *v*_e_ value of the Repeated 150/450 experiment instead of Two-stage GKM, the ratio of *v*_e_ values (1.2: 2.86: 5.58≅0.22: 0.51: 1) is very different from the Evans blue experiment results. This is because when GKM was used in the repeated FUS irradiation experiment, it underestimated the *K*_trans_ and *K*_ep_ values.

Another important aim of this study was to establish a real-time experimental environment in which an ultrasound machine and animals can be placed to get her into the MRI machine. By doing so, when conducting MRI imaging tests, immediate UCA and Gd injection and single or repeated FUS irradiation can be provided simultaneously. The influence of the time delay should be considered for the method used in previous studies (not real-time monitored by MRI). In a study conducted by Park *et al*. [29], under single FUS irradiation, the *K*_trans_ value estimated by DCE-MRI data 0–0.5 hr after FUS irradiation decreased by 60% in the estimation conducted 0–1.5 hr after FUS irradiation. The decreased *K*_trans_ value indicates that the amount of Gd penetration into FUS irradiated brain tissue decreases with time when the BBB is disrupted. This effect may be the result of the gradually restored BBB [29]. On the other hand, the real-time system designed for the present study can prevent the time delay caused by separate operating procedures. Our system can also be used under experimental conditions such as different UCA concentrations and multiple FUS irradiations.

For medications used in the treatment of brain conditions, researchers not only need to know the influences caused by different FUS irradiations, but also need to know the precise estimation of the amount of accumulated drug in the brain. Because brain hemorrhage is likely to be caused by the injection of high doses of UCA [30], [32], [33], the general strategy is to first inject low doses of UCA, followed by gradual increases in the injected UCA doses and multiple FUS irradiations [19], [29], so that the amount of penetrated drug can reach an accumulated amount with planed FUS irradiations. In the future, with the experimental procedures combining the real-time system and repeated FUS irradiations and simultaneous injection of Gd and medications, we will further investigate the penetration of Gd and medications in the rat brain after FUS irradiation.
